# Effectiveness and components of self-management interventions in adult cancer survivors: a protocol for a systematic review and planned meta-analysis

**DOI:** 10.1186/s13643-018-0902-7

**Published:** 2018-12-20

**Authors:** Colleen Ann Cuthbert, Haider H. Samawi, Brenda R. Hemmelgarn, Winson Y. Cheung

**Affiliations:** University of Calgary, Cumming School of Medicine, Department of Oncology, Calgary, Canada

**Keywords:** Self-management, Cancer survivors, Behavior change theory, Intervention components, Systematic review, Protocol

## Abstract

**Background:**

Self-management interventions have been proposed as effective strategies to improve health and well-being and promote optimal coping in cancer survivors. Several reviews have shown benefits of self-management interventions on a variety of patient-reported outcomes. Effective self-management strategies in other chronic disease populations are typically based on theories of behavior change, but the extent of theoretical underpinnings in cancer self-management programs has not been evaluated to date. Our aim is to expand on previous reviews by evaluating the effectiveness of self-management interventions in cancer survivors as well as the theoretical components of such interventions.

**Methods:**

We will conduct a systematic review of self-management interventions for adults who have completed primary treatment for their solid or hematological cancer. Interventions tested using experimental or quasi-experimental methods, with any type of comparator, will be included. A search strategy will be designed with a health sciences librarian and then performed using MEDLINE, EMBASE, PsycINFO, CINAHL, Scopus, the Cochrane database of systematic reviews, the National Institutes of Health clinical trials registry, and the Cochrane CENTRAL registry of controlled trials. Data synthesis will include a narrative and tabular summary of the results. Appropriate statistical analysis may include a meta-analysis using random effects methods to determine the effectiveness of self-management interventions and a meta-regression to evaluate how characteristics of the interventions are associated with the intervention effect. Risk of bias will be evaluated using the Cochrane risk of bias tool or the Risk of Bias in Non-randomized studies tool (RoBANS).

**Discussion:**

The results of this systematic review will add to previous reviews and expand the existing knowledge base of the effectiveness and active components of self-management interventions for adult cancer survivors.

**Systematic review registration:**

PROSPERO CRD42018085300

**Electronic supplementary material:**

The online version of this article (10.1186/s13643-018-0902-7) contains supplementary material, which is available to authorized users.

## Background

There is a global burden of cancer with 18.1 new cases and 9.6 million deaths from cancer expected worldwide in 2018 [1]. The number of people living with a diagnosis of cancer is currently estimated to be over 800,000 in Canada [2], 15.5 million in the USA [3] and 32.6 million worldwide [4]. Due to increased incidence worldwide over the last 15 years [1] and improved survival in high resource countries [1], the number of cancer survivors is expected to grow over the next two decades. Cancer survivors may suffer from a number of long-term physical and psychological health problems as a result of the cancer treatments that they received. Most commonly, these include physical health problems such as chronic fatigue, changes to functional capacity, physical functioning alterations, and body composition changes [5–7]. Psychological health problems may include fear of recurrence, mood and sleep disturbance, and sexuality concerns [5–7]. Research and advocacy work over the last decade [5, 6] has identified the need for health care systems to develop effective strategies for comprehensive survivorship care aimed to help survivors manage their own health. Self-management has been proposed as one such strategy to be used by patients and health care systems to manage the chronic effects of illness [7, 8].

Several recent reviews of self-management interventions in cancer survivors have been conducted [7, 9–12] showing mixed results on the effectiveness of self-management on a variety of outcomes. For example, Kim et al. [9] conducted a systematic review and meta-analysis of self-management interventions in cancer survivors and demonstrated a moderate effect for self-management on quality of life, but no effect on psychological outcomes. They concluded that understanding the components of interventions might lead to more meaningful conclusions about the efficacy of self-management in cancer survivors. Similarly, Boland et al.’s [12] review of six self-management interventions in cancer survivors highlighted the need for more standardized definitions of self-managements and the core components contained therein in order to make a determination of their effectiveness. In addition, in Kim et al.’s [10] systematic review of Internet-based self-management interventions, the authors evaluated whether theories of behavior change were used in self-management interventions, but the specific components of these theories were not detailed nor was a proposed mechanistic link between theory and successful self-management interventions outlined. Finally, a recent thorough examination of the self-management literature in cancer survivors was conducted by Macmillan Cancer Support [13] to produce a policy document. In their summary of findings, it is highlighted that key components of self-management programs should include theoretical foundations. Our goal is to extend previous reviews by including an evaluation of the theoretical basis and specific components of self-management interventions. A thorough evaluation of the theoretical components of self-management interventions for cancer survivors has not been conducted to date. This work is important as it starts to identify the important elements to self-management interventions, addressing not only if they are beneficial, but also what specific components are responsible for the beneficial effects. Thus, the goal of this systematic review is to examine the theories, intervention content, and effectiveness of self-management interventions in adult cancer survivors.

### Objectives

The specific objectives of the review are as follows:

1. To assess the effectiveness of self-management interventions in adult cancer survivors on physical or psychological health outcomes
2. To determine the use/incorporation of behavior change theories and behavior change strategies in self-management interventions for cancer survivors
3. To determine the components of self-management interventions and their effects on physical or psychological health outcomes in adult cancer survivors

## Methods

### Study design

We will conduct a systematic review and planned meta-analysis and possible meta-regression (based on homogeneity of intervention components and outcomes) following the methodology outlined by the Cochrane Handbook of Systematic Reviews [14]. The protocol outlined here follows the Preferred Reporting Items for Systematic review and Meta-analysis Protocols (PRISMA-P) guideline [15]. The PRISMA-P checklist is included in Additional file 1. This protocol is also registered with PROSPERO (registration # CRD42018085300).

### Conceptual framework

#### Self-management

For this systematic review, the relevant research and theoretical literature [7, 16–19] were examined to form a conceptual definition of self-management. The terms self-management and self-care have been used interchangeably in the literature. We chose self-management for this review as it is increasingly recognized that this concept includes a broader conceptualization of the processes, tasks, and outcomes of self-management compared to self-care [7, 19].

Fundamentally, the aim of self-management is to enable and empower patients to achieve optimal health in the context of living with a chronic illness. At the core of self-management is the active participation of patients in their own care, the ability to self-monitor and problem solve, and shared decision-making with health care providers [7, 16]. Lorig et al. [16] outline three tasks that form the core of self-management programs including medical management, role management, and emotional management. In addition, it is recognized that effective self-management programs or interventions should incorporate aspects of both patient education to increase knowledge of their disease, the health care system and resources available to them, and to include theoretically based problem solving and decision-making skills training [7, 16, 18]. For the purposes of this review, our operational definition of self-management is as follows. We considered self-management interventions as those designed to increase patient knowledge about their disease (medical management) and to promote positive coping and adaptation skills (emotional and role management).

#### Cancer survivor

Although the term cancer survivor can be controversial, the most widely adopted definition as outlined by the National Cancer Institute is “an individual is considered a cancer survivor from the time of diagnosis throughout the balance of his or her life” (NIH/NCI) [20]. This broad definition includes the entire cancer trajectory, which encompasses a variety of unique stages, each with differing health concerns and patient needs. Briefly, the phases of cancer survivorship have been described as follows [21]. From the point of diagnosis to the end of primary treatment is the beginning phase of the survivorship trajectory, this phase may be followed by the post-treatment period, and then long-term disease-free survivorship. Some survivors will require ongoing treatment for recurrent or metastatic disease and eventually enter the end of life phase of survivorship. The phase of the cancer trajectory that includes the post-treatment period, sometimes referred to as the re-entry phase [22], is the focus of this review. Thus, our operational definition of survivorship is that a survivor is a patient who has completed primary treatment for their cancer. This phase of cancer survivorship may be considered unique for a number of reasons. After primary treatment is complete, follow-up care may be transitioned from the oncology specialist to other health care providers who may be lacking in cancer-specific expertise [4, 5]. The side effects from cancer treatments will become chronic (versus acute) and require different management strategies [4]. Psychological and social adjustments need to shift to include acceptance of chronic effects of treatment, changes to physical abilities, relationships and work productivity, managing fear of recurrence, finding resources, and navigating the health care system [6, 7]. Management of non-cancerous comorbid medical conditions will need to occur in the context of cancer survivorship. In addition, the re-entry phase represents a critical time for promoting healthy behaviors, positive coping strategies and positive management strategies, to enable survivors to realize long-term health. Thus, self-management needs for those in the re-entry phase of cancer survivorship are unique and the potential benefits of interventions focused on this phase should be evaluated exclusive of interventions targeted to other stages along the cancer trajectory.

#### Behavior change theory

It is increasingly recognized that health behavior change strategies (such as self-management) based on theory are more effective than those lacking a theoretical basis [23, 24]. Bandura argued that, fundamentally, all behavior change theories include the concepts of perceived self-efficacy, self-regulatory skills, knowledge of health and the benefits of health behaviors, outcome expectations, health goals, and perceived barriers and facilitators to change [24]. Effective self-management programs or interventions for a variety of chronic health conditions have included many of the core concepts of behavior change including self-efficacy, self-regulatory skills, tailoring, and disease-specific education [16, 17, 25]. Cancer is increasingly viewed as a chronic disease [5] with growing interest in self-management interventions for cancer survivors [7, 9]. Thus, self-management programs for cancer survivors should be designed with similar theoretical underpinnings as established chronic disease self-management programs.

### Eligibility criteria

Inclusion and exclusion criteria have been formulated using the population, interventions, comparators, outcomes, and design (PICODs) criteria as follows:

- *Population*: Patients diagnosed as an adult (at age 19 or older) with a solid or hematological malignancy who have completed primary treatment for their cancer.
- *Interventions*: Self-management interventions or programs (as defined in the study) delivered in outpatient, inpatient, or community settings.
- *Comparison group*: No treatment control groups (participants receive no treatment whatsoever) [26], wait-list (participants eventually receive the intervention or treatment) [26], attention control groups (participants receive some other attention such as education that is different from the intervention being tested) [26], or standard care control groups (participants receive usual care) [26].
- *Outcomes*: Based on our conceptual definition of self-management and the study objectives, we will consider the following three categories of outcomes: patient-reported outcomes (e.g., measures of physical and psychological health, quality of life, self-efficacy, symptom management, health behavior change), health care outcomes (e.g., health care utilization), or clinical outcomes (e.g., changes to anthropomorphic or fitness measures).
- *Design*: Experimental studies, defined as randomized controlled trials or quasi-experimental (e.g., non-randomized pre-test/post-test) [26].
- *Other eligibility/exclusion*: Studies published in peer-reviewed journals, peer-reviewed abstracts or conference proceedings, or peer-reviewed theses will be included, and no date restriction will be imposed. Only studies published in English will be included. Excluded will be studies with pediatric cancer populations (those diagnosed under the age of 19) [27], interventions comprised of passive educational materials, studies where no outcomes are collected, and reviews of self-management interventions. Grey literature publications that have not undergone peer review (e.g., policy documents, commercial documents) will not be included. Qualitative studies will not be included.

### Information sources and search strategy

An experienced health sciences librarian was consulted to develop the search strategy for this systematic review. A search will be performed using the online databases MEDLINE (Ovid), EMBASE, PsychINFO, CINAHL(EbscoHOST), the Cochrane database of systematic reviews, and Scopus (using a cited reference search based on core articles). The grey literature search will include the Cochrane Central Register for Controlled Trials, the National Institutes of Health clinical trials registry, and the WHO International Clinical Trials Registry Platform. In addition, conference abstracts will be read to identify any potential studies. The grey literature will help to identify any studies not identified through other search strategies. However, only those studies identified from the grey literature search that have also been peer-reviewed will be included. The search will be developed using medical subject headings (MeSH), keywords, title words, or abstract words related to self-management, self-care, cancer survivor, cancer patient, controlled trials, or experimental studies. A draft of the MEDLINE search strategy is outlined in Additional file 2. After the MEDLINE search strategy is completed, a random sample of 50 titles and abstracts will be evaluated to ensure previously identified self-management studies are captured. The search strategy will then be refined as needed, and the final search strategy will be used as a template for the other databases. After the database searches are complete, handsearching the reference sections from any identified studies will be reviewed using backward citation searching to identify any additional studies. Systematic reviews of self-management interventions will be read to identify additional studies.

### Study records

#### Data management

The searched results will be managed using Endnote reference management software (version X7).

### Selection process

Using Endnote, search results will be merged and duplicates removed. Duplicates will be first identified in Endnote using the find duplicates function. All duplicates will be reviewed and any duplicate of a study with the same author and year will be removed. Any study that has the same author and title but different dates (indicating a potential study update, or pilot data) will be retained for further review. We will use a two-step process for the selection of articles yielded from our search to be included in the systematic review. Primary screening will be conducted independently by two reviewers (CC and HS) and will include screening of titles and abstracts against the inclusion criteria (see primary screening tool Additional file 3). Any article where the reviewers are unclear based on title or because there is no abstract will be retained. Reviewer agreement for primary screening will be measured using Cohen’s kappa, and agreement greater than 0.75 [14] will be considered excellent agreement. Agreement level below this cut-point will trigger a re-evaluation of the primary screening tool. Secondary screening will be conducted independently by the same two reviewers to evaluate full-text articles against the inclusion criteria (see secondary screening tool Additional file 4). The reviewers will not be blinded to the authors, institutions, or journal of publication. Disagreements between reviewers will be resolved through discussion or third-party reviewer, if consensus can not be reached. All reasons for exclusion of a study will be recorded.

### Data collection process

#### Data abstraction

A standardized data abstraction form will be used for each study and will be completed independently by two reviewers (CC and HS). Information to be abstracted from the studies will include study characteristics (study design, study population, methods, data analysis, and results). The theoretical basis for studies and self-management intervention components will be abstracted using a standardized form that was compiled following the Template for Intervention Description and Replication (TIDier) checklist [28] and based on similar systematic reviews [29, 30] and the self-management literature [7, 16, 24, 25]. Items will broadly include (1) whether theoretical basis for intervention is explained, (2) duration and intensity of the intervention, (3) was the intervention tailored to a specific group, (3) how was the interventionist trained, (4) what educational content was included, (5) what behavioral change skills were taught, (6) what illness adjustment skills were taught, and (7) was the intervention structured to support behavior change. The data abstraction form is outlined in detail in Additional file 5. Outcomes to be abstracted will broadly fall into patient-reported outcomes, clinical outcomes, or health care outcomes. Outcome definitions may be further refined during the review process based on outcomes in the included studies. We will attempt to contact study authors to obtain missing or incomplete information related to intervention components or outcome data. The data abstraction form will be pilot tested on 10% of the studies by each reviewer to ensure agreement. To evaluate the possibility of risk of bias in the studies, we will use the Cochrane Collaboration Risk of Bias tool [14] for randomized studies. For non-randomized studies, such as pre-test/post-test designs, the Risk of Bias of Non-randomized studies (RoBANs) [31] will be used to determine the risk of bias. Two reviewers (CC and HS) will complete the bias assessment independently. Any disagreement will be resolved through consultation with the third and fourth authors (BH and WC). Domains evaluated for randomized studies according to the Cochrane risk of bias tool will include selection bias, performance bias, detection bias, attrition bias, and reporting bias. For non-randomized studies, the risk of bias domains include the selection of participants, confounding variables, the measurement of exposure, the blinding of the outcome assessments, incomplete outcome data, and selective outcome reporting. Each domain will be rated independently by each reviewer as having high, low, or unclear risk of bias.

#### Assessing the quality of the evidence

We will use the Grading of Recommendations Assessment, Development and Evaluation (GRADE) framework to evaluate the overall quality of the body of the evidence [32] from this review. Each outcome will be evaluated and assigned a rating of either high, moderate, low, or very low quality of evidence. Two reviewers (CC, HS) will independently determine the rating for the quality of evidence according to criteria outlined by the GRADE working group [32]. Any disagreement of the rating will be resolved by including a third reviewer (WC).

### Data synthesis

The process for selection of studies included in the review will be represented in a PRISMA flow diagram [15]. The data will be synthesized and presented in narrative and table format. Study characteristics, intervention details, and risk of bias assessment will be presented in table format. The table format will follow similar structure and content of the data abstraction forms, and where possible, the magnitude of effect on measured outcomes will be included. Details of the self-management theories and components will be synthesized in a table. Study outcomes will be presented in narrative and table form and grouped according to patient reported, health care, or clinical outcomes.

### Data analysis

We expect heterogeneity of intervention components and outcomes, which may not yield sufficient data for statistical analysis across all measures or endpoints. Clinical and statistical heterogeneity will be assessed by the review team. We will conduct a meta-analysis only if at least ten studies are sufficiently homogeneous in terms of participants, interventions, and outcomes as outlined by the Cochrane collaboration [14]. If there is a sufficient number of studies and adequate homogeneity of specific outcomes, a meta-analysis will be performed on these outcomes to determine the direction and size of the pooled treatment effect of self-management interventions. To determine our measurement of treatment effect, we will assess the type of data reported. We anticipate that effects sizes or mean differences will be reported for most outcome variables. For continuous data, we will calculate standard mean difference, and for dichotomous outcomes, we will calculate risk ratio. We do not anticipate time to event data or ordinal data for self-management interventions. We will include a summary table of the intervention effect estimate, the *p* value and confidence interval for the studies included in the meta-analysis. Consultation with a statistician will occur in order to determine if appropriate statistical methods were used to estimate effects in cluster randomized trials and also to correct for the level of analysis if required. In addition, for trials with multiple interventions or treatment groups, we will only include comparisons between the self-management intervention and usual care (i.e., no treatment control group as self-management is not a standard of care).

Meta-analysis will be performed using RevMan [14] with a random effects model as this is recommended for quantitative data synthesis of studies with heterogeneity [14]. A forest plot will be used to present the summary of findings from the meta-analysis. A subgroup analysis, or meta-regression, will be performed if ten or more studies are included in the meta-analysis [14]. Meta-regression will be used to determine how the characteristics of the intervention (behavior change theory, behavior change skills, disease-specific educational content, behavior change support, illness adjustment skills, or intervention length or intensity) are associated with the intervention effect. The *Q* statistic and *I* statistic will be respectively used to describe the presence and degree of heterogeneity [14]. A funnel plot and the Begg’s and Egger’s tests will be used to examine the presence of publication bias [14].

## Discussion

This systematic review will build on previous reviews on self-management interventions in adult cancer survivors. Our aim for this systematic review is to summarize the existing evidence in this area and to deconstruct the component parts, or “active ingredients”, of self-management interventions. With cancer care systems striving toward providing more comprehensive survivorship care, self-management has the potential to be an important strategy to meet this goal. However, to date, self-management interventions have been heterogeneous in their design and targeted outcomes. This systematic review may provide a more detailed understanding of how to design self-management interventions to realize the most benefit for cancer survivors. The findings from this review may inform future research on theoretical considerations and outcome measures when designing tailored self-management interventions for cancer survivors. In addition, this review may provide information to help design self-management programs offered to cancer survivors through cancer care systems. This review may be limited to a narrative synthesis of the evidence rather than a statistical analysis. We believe that despite this potential limitation, the review will still fill an important knowledge gap regarding self-management interventions in cancer survivors.

## Additional files

Additional file 1: PRISMA-P checklist as required for submission. (DOCX 20 kb)

Additional file 2: Draft search strategy for MEDLINE. (DOCX 12 kb)

Additional file 3: Primary screening tool for titles and abstracts. (DOCX 14 kb)

Additional file 4: Secondary screening tool for full text articles. (DOCX 14 kb)

Additional file 5: Data abstraction tool. (DOCX 14 kb)

## Abbreviations

PICODs: Patient, intervention, comparison, outcomes, design
PRISMA-P: Preferred Reporting Items for Systematic review and Meta-analysis Protocols
RoBANS: Risk of Bias in Non-randomized studies tool
TIDier: Template for Intervention Description and Replication
