# Supplementary material for: Effectiveness and components of self-management interventions in adult cancer survivors: a protocol for a systematic review and planned meta-analysis
Source: Syst Rev. 2018 Dec 20;7:238. doi: 10.1186/s13643-018-0902-7 (PMC6300917; doi:10.1186/s13643-018-0902-7)
Supplement: Supplementary file 2 — Draft search strategy for MEDLINE. (DOCX 12 kb) [file 13643_2018_902_MOESM2_ESM.docx]

Additional file 2

Draft Search Strategy for MEDLINE

| 1. exp Neoplasms/ |
| --- |
| 2. exp Patients/ |
| 3. Survivors/ |
| 4. (cancer* or neoplasm*).tw,kw. |
| 5. (patient* or survivor*).tw,kw. |
| 6. 1 or 4 |
| 7. 2 or 3 or 5 |
| 8. 6 and 7 |
| 9. self care/ |
| 10. (self care or self help or self guided or self directed or self manage or self regulate*).tw,kw. |
| 11. (psychoeducational or psychoeducational intervention*).tw,kw. |
| 12. (patient directed or patient guided or patient managed).tw,kw. |
| 13. 9 or 10 or 11 or 12 |
| 14. 8 and 13 |
| 15. (controlled clinical trial or randomized controlled trial) |
| 16. (groups or placebo* or random* or trial*).tw. |
| 17. intervention.mp. |
| 18. (pre test or pretest or post test or posttest).tw |
| 19. 15 or 16 or 17 or 18 |
| 20. 14 and 19 |
| 21. limit 20 to "all adult (19 plus years)" |
| 22. limit 20 to ("newborn infant (birth to 1 month)" or "infant (1 to 23 months)" or "preschool child (2 to 5 years)" or "child (6 to 12 years)") |
| 23. 21 and 22 |
| 24. 22 not 23 |
| 25. 20 not 24 |
