# Supplementary material for: Effectiveness and components of self-management interventions in adult cancer survivors: a protocol for a systematic review and planned meta-analysis
Source: Syst Rev. 2018 Dec 20;7:238. doi: 10.1186/s13643-018-0902-7 (PMC6300917; doi:10.1186/s13643-018-0902-7)
Supplement: Supplementary file 3 — Primary screening tool for titles and abstracts. (DOCX 14 kb) [file 13643_2018_902_MOESM3_ESM.docx]

**Additional file 3**

**Primary Screening: Titles and Abstracts**

Include study for full text review if the following criteria are met.

|  | Yes | No | Unsure* |
| --- | --- | --- | --- |
| 1. Is the article peer reviewed |  |  |  |
| 2. Population (if all yes or unsure include)   1. Adults > 18 years 2. Cancer Patients (solid or hematological malignancies) 3. Completed cancer treatment   Exclude: (any yes exclude)   - Under 18 years of age - Patients undergoing active cancer treatment - Patients undergoing cancer screening |  |  |  |
| 3. Intervention (if all yes or unsure include)   1. Identified as self-management (or related term including self-care, self-help, or psychoeducational) 2. Described as program, intervention, tool, or strategy   Exclude: (any yes exclude)   - Passive educational material only - Psychological counselling only |  |  |  |
| 4. Study Design (all yes or unsure include)   1. Experimental studies including randomized controlled trial (with any comparators) and quasi-experimental.   Exclude: (any yes exclude)   - Non-experimental studies (cohort, case control, case report) - Literature or systematic reviews** - Clinical practice guidelines or outlines of models of care |  |  |  |
| 5. Outcomes (if all yes or unsure include)   1. Any outcome is measured (patient reported, clinical or health services)   Exclude: (any yes exclude)   - No outcome measured |  |  |  |
| Other: no date restrictions, no restrictions on length of time of intervention, no language restrictions, no setting restrictions |  |  |  |

*if unsure, articles should be retained for full text review

** literature and systematic reviews will be read to identify any additional studies to be included.
