# Supplementary material for: Effectiveness and components of self-management interventions in adult cancer survivors: a protocol for a systematic review and planned meta-analysis
Source: Syst Rev. 2018 Dec 20;7:238. doi: 10.1186/s13643-018-0902-7 (PMC6300917; doi:10.1186/s13643-018-0902-7)
Supplement: Supplementary file 4 — Secondary screening tool for full text articles. (DOCX 14 kb) [file 13643_2018_902_MOESM4_ESM.docx]

**Additional file 4**

**Secondary Screening: Full text articles**

|  | Yes | No |
| --- | --- | --- |
| 1. Population((if all yes include)   1. Adults > 18 years 2. Cancer Patients (solid or hematological malignancies) 3. Completed cancer treatment   Exclude: (any yes exclude)   - Under 18 years of age - Patients undergoing active cancer treatment - Patients undergoing cancer screening |  |  |
| 2. Intervention (if all yes include)   1. Identified as self-management (or related term including self-care, self-help, or psychoeducational) 2. Described as program, intervention, tool, or strategy   Exclude: (any yes exclude)   - Passive educational material only - Psychological counselling only |  |  |
| 3. Study Design (if all yes include)   1. Experimental studies including randomized controlled trial (with any comparators) and quasi-experimental.   Exclude: (any yes exclude)   - Non-experimental studies (cohort, case control, case report) - Literature or systematic reviews** - Clinical practice guidelines or outlines of models of care |  |  |
| 4. Outcomes (if all yes include)   1. Any outcome is measured (patient reported, clinical or health services)   Exclude: (any yes exclude)   - No outcome measured |  |  |
| If excluded for any reason, indicate why |  |  |
