# Supplementary material for: Effectiveness and components of self-management interventions in adult cancer survivors: a protocol for a systematic review and planned meta-analysis
Source: Syst Rev. 2018 Dec 20;7:238. doi: 10.1186/s13643-018-0902-7 (PMC6300917; doi:10.1186/s13643-018-0902-7)
Supplement: Supplementary file 5 — Data abstraction tool. (DOCX 14 kb) [file 13643_2018_902_MOESM5_ESM.docx]

**Additional file 5**

Data Abstraction Tool

| **Reviewer** | **Article Number** | **Title of Article** |
| --- | --- | --- |
| **Authors** | **Journal, volume, issue** | **Year Published/Dates of Study** |
| **Country of Origin** | **Language** | **Study Objective** |
|  | **Intervention** | **Control** |
| **Study Population** |  |  |
| Number of Participants |  |  |
| Mean Age |  |  |
| Gender |  |  |
| Ethnicity |  |  |
| Socioeconomic status (income and education) |  |  |
| Cancer type and duration post treatment |  |  |
| Other clinical characteristics (comorbidities, stage of disease, time since diagnosis) |  |  |
| **Study Setting**  (where intervention provided e.g. home, clinic, community) |  |  |
| **Methods** | | |
| Design of study (e.g. RCT, pre-test post-test) |  |  |
| Inclusion and exclusion criteria |  |  |
| Randomization procedure explained |  |  |
| Blinding |  |  |
| Duration of follow-up |  |  |
| Recruitment methods and period |  |  |
| Completion rates |  |  |
| Sample size |  |  |
| **Intervention** | | |
| Description of intervention |  |  |
| Control condition |  |  |
| Format/mode of delivery |  |  |
| Retention rates |  |  |
| Adherence rates |  |  |
| Modifications during intervention |  |  |
| **Outcomes** | | |
| Type of outcomes measured (e.g. patient reported, clinical, health care utilization) and unit of measure |  |  |
| Timing of measurements |  |  |
| Intention to treat analysis |  |  |
| Adverse outcomes |  |  |
| **Results** | | |
| Summary data for each group and outcomes measured (means, sd, effect size) |  |  |
| Correspondence with authors required (Y/N) |  | |

**Data Abstraction – Self-Management Intervention Components**

| Intervention intensity (number of contacts between interventionist and patient over study) |  |
| --- | --- |
| Duration of intervention (in months) |  |
| Theory of behavior change identified (Y/N, if yes specify theory) |  |
| Was intervention tailored to specific patient group (Y/N) |  |
| Standardized Training of Interventionist (Y/N) |  |
| Educational Content delivered |  |
| Content specific to the disease (e.g. general information about cancer) (Y/N) |  |
| Content specific to healthy behaviors (e.g. nutrition, physical activity, stress reduction) (Y/N) |  |
| Content specific to management of symptoms (e.g. fatigue) (Y/N) |  |
| Behavior Change Skills Taught |  |
| Self-monitoring (through logs, journals etc) (Y/N) |  |
| Goal setting skills taught (Y/N) |  |
| Problem solving skills taught (Y/N) |  |
| Decision making skills taught (Y/N) |  |
| Maintenance strategies taught Y/N) |  |
| Illness Adjustment Skills |  |
| Management of psychological aspects of living with cancer taught (Y/N) |  |
| Management of physical aspects of living with cancer taught (Y/N) |  |
| Communication with health care providers (Y/N) |  |
| Intervention Structured to Support Behavior Change |  |
| Peer/group interaction (Y/N) |  |
| Reinforcement/rewards of goals |  |
